# *VHL* missense mutations in the p53 binding domain show different effects on p53 signaling and HIFα degradation in clear cell renal cell carcinoma

**DOI:** 10.18632/oncotarget.14372

**Published:** 2016-12-30

**Authors:** Caroline Fanja Razafinjatovo, Daniel Stiehl, Eva Deininger, Markus Rechsteiner, Holger Moch, Peter Schraml

**Affiliations:** ^1^ Department of Pathology and Molecular Pathology, University Hospital Zurich, Zurich, Switzerland; ^2^ Institute of Physiology and Zurich Center for Integrative Human Physiology (ZIHP), University of Zurich, Zurich, Switzerland

**Keywords:** clear cell renal cell carcinoma, *VHL* missense mutations, pVHL binding sites, p53, HIFα

## Abstract

Clear cell Renal Cell Carcinoma (ccRCC) formation is connected to functional loss of the von Hippel-Lindau (*VHL*) gene. Recent data identified its gene product, pVHL, as a multifunctional adaptor protein which interacts with HIFα subunits but also with the tumor suppressor p53. p53 is hardly expressed and rarely mutated in most ccRCC. We showed that low and absent p53 expression correlated with the severity of *VHL* mutations in 262 analyzed ccRCC tissues. In contrast to nonsense and frameshift mutations which abrogate virtually all pVHL functions, missense mutations may rather influence one or few functions. Therefore, we focused on four *VHL* missense mutations, which affect the overlapping pVHL binding sites of p53 and Elongin C, by investigating their impact on HIFα degradation, p53 expression and signaling, as well as on cellular behavior using ccRCC cell lines and tissues. *TP53* mRNA and its effector targets *p21*, *Bax* and *Noxa*, were altered both in engineered cell lines and in tumor tissues which carried the same missense mutations. Two of these mutations were not able to degrade HIFα whereas the remaining two mutations led to HIFα downregulation, suggesting the latter are p53 binding site-specific. The selected *VHL* missense mutations further enhanced tumor cell survival, but had no effects on cell proliferation. Whereas Sunitinib was able to efficiently reduce cell proliferation, Camptothecin was additionally able to increase apoptotic activity of the tumor cells. It is concluded that systematic characterization of the *VHL* mutation status may help optimizing targeted therapy for patients with metastatic ccRCC.

## INTRODUCTION

Renal cell carcinoma (RCC) is one of the most common cancer types worldwide with clear cell RCC (ccRCC) being the most frequent and aggressive RCC subtype [1, 2]. In ccRCC the von Hippel-Lindau tumor suppressor gene (*VHL*) is frequently altered by deletion of one allele (90%) and promoter methylation (up to 20%) or mutations (70-80%) of the other. *VHL* inactivation is considered as a critical part of tumor initiation [3–5]. In addition to its well-known function as E3 ubiquitin ligase for ubiquitination and proteasomal degradation of hypoxia-inducible factor subunits (HIF1α and HIF2α) [6–8], the *VHL* protein (pVHL) has been recently identified as a multiadaptor protein involved in a variety of cellular processes such as microtubule stability, activation of p53, neuronal apoptosis, cellular senescence and aneuploidy, ubiquitination of RNA polymerase II and regulation of NFkB activity [1].

Given different types of *VHL* mutations, a deeper insight in the biological effects of *VHL* mutations may allow a better prediction of ccRCC prognosis. In particular, *VHL* loss-of-function mutations (LOF) (frameshift, nonsense and splice site mutations) highly likely abrogate pVHL function, whereas the consequences of missense mutations on pVHL stability and target binding ability are rather unclear. Missense mutations may provoke diverse effects on pVHL interactions with binding partners, thus exerting different impact on pathways normally regulated by pVHL. This was shown for HIF1α and HIF2α degradation [9] as well as for other pVHL binding partners, including Jade1, RPB1, VDU1, EEF1A1 and CCT-ζ-2, for which loss of binding capability upon missense mutations was demonstrated [10–15].

p53 is a well-known tumor suppressor gene, whose activation by hypoxia or DNA damage leads to cell cycle arrest, DNA repair and apoptosis. Under cellular stress, p53 level is increased by inhibition of its interaction with MDM2 and activated by post-translational modifications through different regulators which lead to transactivation of its downstream target genes *p21* (alias *CDKN1A*, growth arrest), *Bax* and *Noxa* (apoptosis) [16]. The role of p53 in ccRCC and its relation to pVHL is yet unclear. Two previous studies showed that pVHL can stabilize p53 and enhance its transcriptional activity [17, 18] whereas another study found that p53 expression is not pVHL-dependent [19]. In addition, pVHL inactivation in RCC cells lead to decreased apoptosis [20], which may be explained by the lack of phosphorylation of pVHL by checkpoint-kinase 2, impairing the recruitment of p53 coactivators (such as p300 and Tip60) [21].

Tumors with p53 mutations are known to be associated with chemoresistance [22]. p53 is one of the most frequently mutated genes in several cancers [23], but p53 mutations are rare in ccRCC [24–26]. Interestingly, ccRCC is resistant to chemotherapy and Gurova et al. suggested that p53 signaling is repressed by mechanisms independent of p53 mutations [27]. ccRCC is currently treated with anti-angiogenic drugs, such as the Tyrosine-Kinase-Inhibitors (TKI) Sorafenib and Sunitinib, to counter the effects of the HIF1/2α accumulation occurring upon pVHL inactivation. The efficiency of this therapeutic strategy is still suboptimal [28]. As shown for colorectal cancer where p53 negative cells were less responsive to anti-angiogenic treatment than wild-type p53 cells [29], alteration of p53 signaling may also be an explanation for the low response rate in ccRCC.

We hypothesized that *VHL* missense mutations occurring in the p53 binding domain of pVHL lead to deficient p53 transactivation and/or promote HIF1α and HIF2α accumulation, thus impacting tumor behavior and response to treatment. In this study, we investigated four different missense mutations located in the p53 binding site (codons 154-163), which is overlapping with the ElonginC binding domain (codons 157-171). Due to this overlap, the missense mutations investigated could have an impact on p53 signaling and/or on HIF1/2α degradation through an altered binding to ElonginC. Our goal was to evaluate the selected missense mutations effectiveness in HIF1/2α degradation, p53 transactivation, and their response to chemotherapy and TKI.

## RESULTS

### Expression of p53 in ccRCC

Pathological and molecular characteristics of renal cell cancers on the tissue microarray (TMA) were previously described [9, 30]. In brief, the TMA consisted of 262 clear cell, 48 papillary (24 type I, 24 type II), 15 chromophobe RCC, eight non specified RCC, 22 oncocytoma and 28 normal tissue cores. 181 of 262 ccRCC were *VHL* mutated (69%).

**Figure 1:**
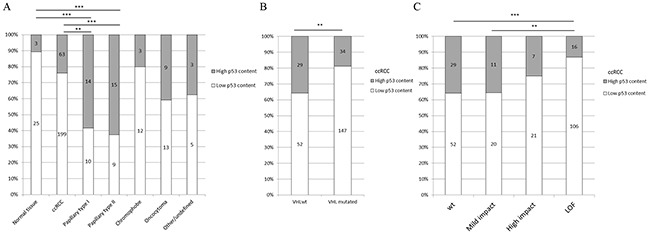
**A.** p53 protein expression in RCC tissue; **B.** p53 protein expression in ccRCC and *VHL* mutation status; and **C.** p53 protein expression in ccRCC and mutation impact on pVHL stability. Wt: *VHL* wild type; Mild impact: Destabilizing or neutral or stabilizing; High impact: highly stabilizing or highly destabilizing; LOF (Loss of Function): Frameshift and nonsense mutations. p-value: *<0.05, **<0.01, ***<0.001

TMA analysis revealed absent or only low p53 expression in most ccRCC (76%), and chromophobe RCC (80%), whereas nuclear p53 positivity was high in about 60% of papillary RCC (Figure 1A). By separating the 262 ccRCC in *VHL* wild-type and *VHL* mutated tumors, we observed that p53 expression was less frequent in tumors with *VHL* alterations (p=0.0212) (Figure 1B). Notably, the p53 expression frequency decreased with the predicted impact of mutations on the function of pVHL (Figure 1C).

### Selection of mutations in the p53/EloC binding domains of pVHL

The binding domains of p53 and EloC are located between codons 154 to 163 and 157 to 171, respectively [34]. Among 254 *VHL* mutations found in 360 ccRCC tissue specimen (70.6%), 25 (9.8%) resided in these binding domains. Eleven (4.3%) were missense mutations causing an amino acid exchange. Eight missense mutations were predicted to destabilize pVHL and three missense mutations had no or little impact on pVHL stability.

For this study, we selected four out of 11 missense mutations occurring in the p53/EloC binding domain for further analysis. Three were predicted to have mild impact on pVHL (Leu158Val, Arg161Gln, and Cys162Arg) and one was predicted to highly destabilize pVHL (Arg161Pro). Two other missense mutations occurring in the HIF1/2α binding domain (Tyr98His, Tyr98Asn), as well as one nonsense mutation located in the p53/EloC binding domain (Arg161X) were predicted to differently impair HIF1/2α degradation pathway and were used as controls for the cell line experiments (summarized in Table 1).

**Table 1:** Cell lines with *VHL*-expressing vector constructs, predicted effects on pVHL stability and affected binding domains of pVHL

RCC4 cell lines stably expressing:	Predicted effect	Binding domain affected
Babe (Ser65Trp)	destabilizing and cause protein malfunction	others
VHL30	none	none
Leu158Val	slightly destabilizing	p53/EloC
Arg161Gln	neutral	p53/EloC
Cys162Arg	neutral	p53/EloC
Arg161Pro	destabilizing and cause protein malfunction	p53/EloC
Tyr98His	neutral	HIF1/2α
Tyr98Asn	destabilizing	HIF1/2α
Arg161X	Loss of function	HIF1/2α/p53/EloC

### Effects of selected *VHL* mutations on HIF1/2α and p53

**Figure 2:**
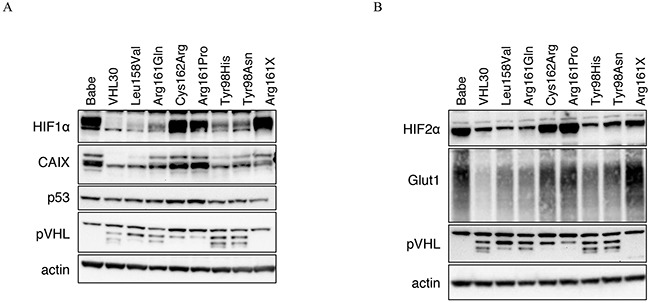
**A.** and **B.** Western blots showing pVHL, p53, HIF1/2α and HIF1/2α targets expression (CAIX and Glut1) in the established stable cell lines.

The established stable cell lines were investigated by Western Blot for the effects of the *VHL* missense mutations on HIF1/2α degradation and the two HIF1/2α targets CAIX and Glut1 (Figure 2). As expected, pVHL was undetectable in RCC4 Babe and RCC4 Arg161X. HIF1α and 2α were stabilized in RCC4 Babe, Arg161Pro, Arg161X, and also in RCC4 Cys162Arg. RCC4 Arg161Gln, Tyr98His and Tyr98Asn showed partial HIF1α and 2α degradation. HIF1/2α strong degradation was seen in Leu158Val which was similar to RCC4 VHL30 (wild-type). CAIX and Glut1 expression correlated with HIF1/2α. No clear difference in p53 expression was seen between *VHL* wild-type and mutated cell lines.

### Impact of *VHL* mutations on p53 downstream targets in cell lines

**Figure 3:**
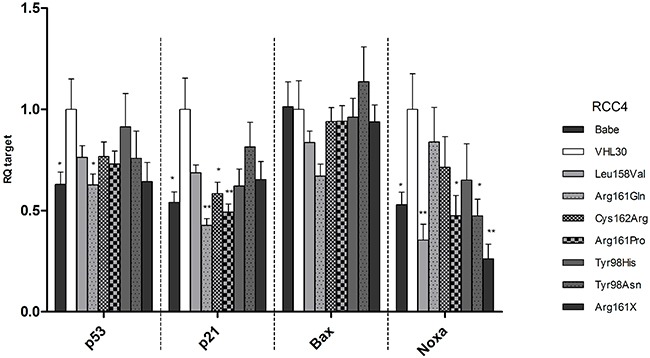
mRNA levels of TP53, p21, Bax, and Noxa in the established RCC4 stable cell lines For each gene the mRNA level of the VHL30 sample was used as reference and compared to the mRNA levels in the other cell lines. p-value: *<0.05, **<0.01 (data are presented as mean +SEM).

Previous studies showed that pVHL enhances p53 transcriptional activity which was reduced by Ser111Arg or Ser111Cys missense mutations [19, 23]. Here we studied the effects of our selected *VHL* mutations on the RNA level of *TP53* and its effectors *p21*, *Bax* and *Noxa*. The transcription levels of *TP53*, *p21* and *Noxa* in cells expressing the different mutant forms were generally lower compared to the wild-type form of pVHL (Figure 3). Transcription levels of *p21* and *Noxa* were significantly reduced in most *VHL* mutants compared to RCC4 expressing *VHL* wild-type. *Bax* RNA levels were lower only in RCC4 Leu158Val and Arg161Gln. Among the missense mutations in the p53 binding domain, no significant difference in RNA levels was seen between the three non-destabilizing/slightly destabilizing mutants and the one with destabilizing effects on pVHL.

### Impact of *VHL* mutations on p53 downstream targets in tissues

**Figure 4:**
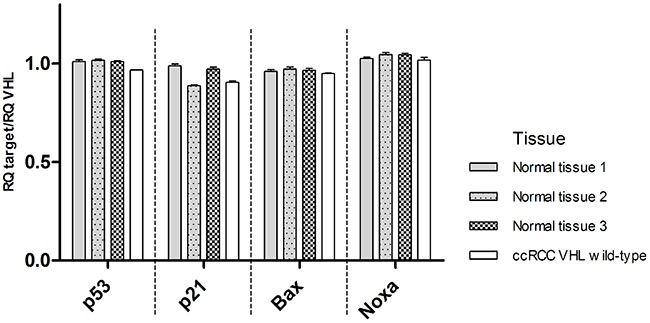
mRNA levels of TP53, p21, Bax, Noxa relative to VHL transcription levels in three normal kidney tissues and one VHL wild-type ccRCC (data are presented as mean +SEM)

**Figure 5:**
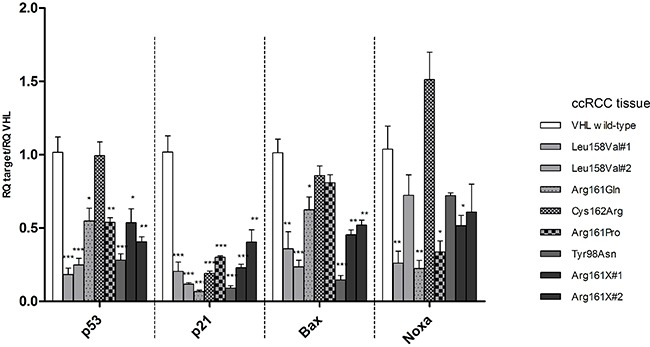
mRNA levels of *TP53, p21, Bax, Noxa* relative to *VHL* wild type transcription levels in ccRCC tissue samples carrying the *VHL* mutations selected for cell line experiments No tissue with *VHL* mutation Ser65Trp (endogenous mutation of RCC4) and Tyr98His was available in our cohort. For each target the mRNA level of the *VHL* wild-type tumor is used as reference and compared to the mRNA levels in the other tumors. p-value: *<0.05, **<0.01, ***<0.001 (data are presented as mean +SEM)

Nine ccRCC tissues (one *VHL* wild-type, two with mutation Leu158Val (#1, #2), one with Arg161Gln, one with Cys162Arg, one with Arg161Pro, one with Tyr98Asn and two with Arg161X (#1, #2)) and three normal kidney tissues were investigated for RNA expression of *VHL*, *TP53* and its downstream targets. *TP53*, *p21*, *Bax* and *Noxa* RNA levels relative to *VHL* were compared in three normal tissues and in one *VHL* wild-type ccRCC. *TP53*, *p21*, *Bax* and *Noxa* expression levels in tumor tissue were similar to the ones seen in normal tissues (Figure 4). The RNA levels of *TP53* and its downstream targets relative to *VHL* transcription levels were assessed in ccRCC samples expressing the mutant forms of *VHL* (Figure 5). Similarly to the established cell lines, wild-type *VHL* tumor showed generally higher RNA levels of *TP53* and its downstream targets than *VHL* mutated tumors. All eight mutant samples showed a decrease of *p21, Bax and Noxa* RNA levels compared to *VHL* wild-type except for *Noxa* in the Cys162Arg mutated tumor. *VHL* missense mutations located in the p53 binding domain and *VHL* mutations in the HIF1/2α binding domain were similarly affected in p53 signaling. Notably, no significant differences in the RNA levels were observed between the mutation Arg161Pro which highly destabilize pVHL and the other mutations which are predicted to have a lower impact on pVHL stability.

### Influence of *HIF1α* on p53 signaling

**Figure 6:**
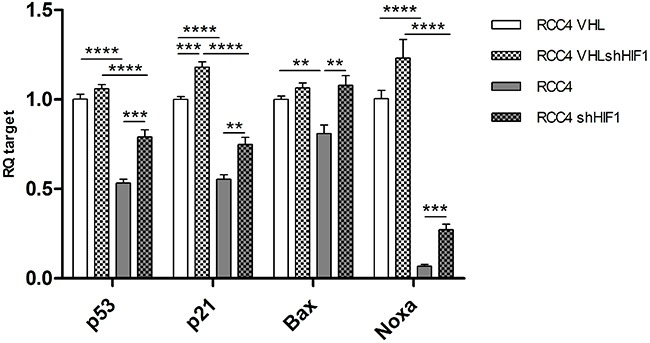
mRNA levels of *TP53, p21, Bax* and *Noxa* in RCC4 expressing *VHL* (white), *VHL* deficient (grey), and corresponding *HIF1α* knockdown (patterned) p-value: *<0.05, **<0.01, ***<0.001, ****<0.0001 (data are presented as mean +SEM).

Since it is known that HIF1α can negatively regulate p53 activity [31, 32], we next asked whether impaired p53 signaling was directly linked to *VHL* mutation status or was a consequence of the deregulated pVHL/HIF1α axis. For this purpose, RCC4 cells with re-expressing *VHL* wild-type and with deficient *VHL* were transfected with a small hairpin RNA for *HIF1α* knockdown (Figure 6). *HIF1α* knockdown led to upregulation of *TP53*, *p21*, *Bax* and *Noxa* transcription in *VHL* wild-type and *VHL* deficient cell lines. This effect was significant for all targets in the absence of functional pVHL and no or little difference was seen in *VHL* expressing cell lines. Cell lines expressing functional pVHL showed higher RNA levels for all targets compared to pVHL deficient cell lines. These results suggest that the functional integrity of pVHL has a dominating impact on p53 signaling.

### p21 expression in ccRCC tumor tissues

**Figure 7:**
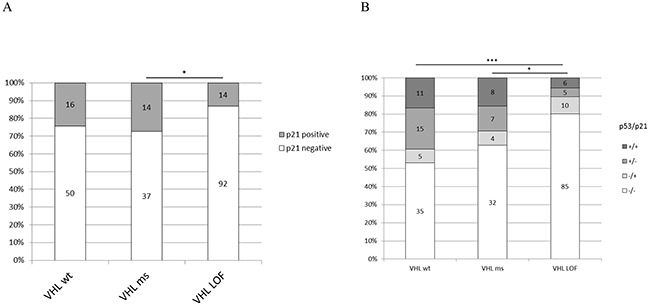
**A.** p21 protein expression in ccRCC tissue and VHL mutation type; **B.** p53/p21 combined expression and *VHL* mutation type. Wt: wild-type, ms: missense, LOF: Loss of function. p-value: *<0.05, **<0.01, ***<0.001

The RCC TMA which was analyzed for p53 expression was also immunostained against p21 to correlate p21 expression with the *VHL* mutation status and p53 expression. ccRCC with LOF mutations expressed less p21 than *VHL* wild-type tumors (not significant) and missense mutated tumors (p<0.05) (Figure 7A). About 10% of the p53 negative and 52% of the p53 positive cores showed a high content of p21 (p<0.0001). The expression pattern of p53 and p21 is significantly different in *VHL* LOF ccRCC compared to missense (p=0.0288) and wild-type cases (p<0.0001) (Figure 7B). The tumors positive for both p53 and p21 were less frequent in *VHL* LOF ccRCC than in wild-type cases (p=0.0327).

### Effects of p53 and HIF1/2α binding site-specific *VHL* mutations on apoptosis and cell proliferation

**Figure 8:**
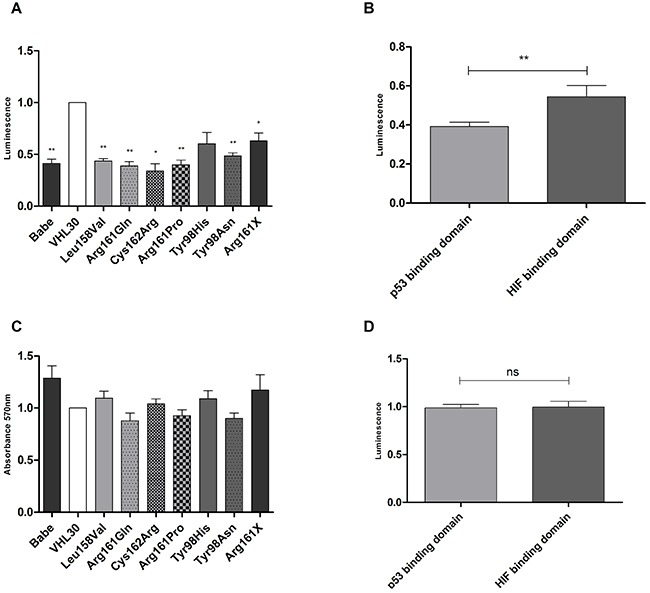
Effects of *VHL* mutations on apoptosis and cell proliferation **A.** Apoptotic behavior of *VHL* wild type and mutated RCC4 cells; **B.** apoptotic behavior of RCC4 with missense mutations in the p53 and HIF1/2α binding domains; **C.** Proliferative behavior of *VHL* wild type and mutated RCC4 cells; D: Proliferative behavior of RCC4 with missense mutations in the p53 and HIF1/2α binding domains. *VHL*30 expression was used as reference and compared to the other cell lines. p-value: *<0.05, **<0.01 (data are presented as mean +SEM).

The apoptotic behavior of cells expressing different *VHL* missense mutations was evaluated by Caspase 3/7 assay. All pVHL mutants were deficient in apoptosis compared to *VHL* wild- type (Figure 8A). Cells with p53 binding site-specific *VHL* mutations showed significantly lower apoptotic activity than those with mutations that affected the HIF1/2α binding domain (p=0.0088) (Figure 8B). In contrast, cell proliferation was not influenced by the different binding site-specific *VHL* mutants (Figure 8C-8D).

### Apoptotic and proliferative behavior of cells upon treatment with Camptothecin and/or Sunitinib

**Figure 9:**
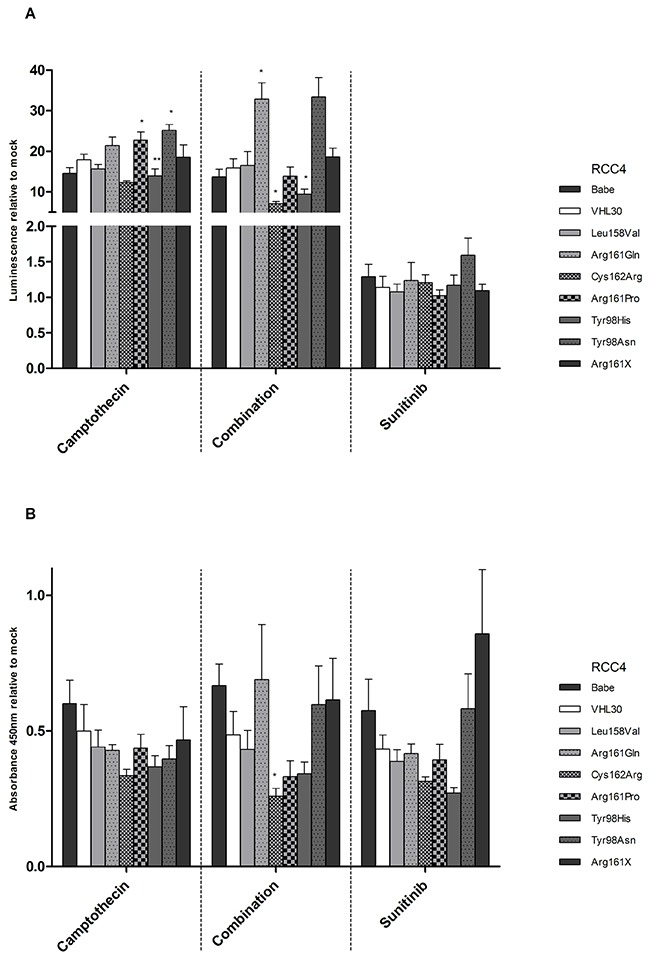
**A.** Apoptotic and **B.** proliferative behavior of *VHL* wt and mutated RCC4 cells after treatment with Camptothecin, Camptothecin and Sunitinib (Combination), and Sunitinib. Signals after treatment of each cell line were normalized with corresponding mock signals (without treatment). *VHL*30 was used as reference and compared to the other cell lines. p-value: *<0.05, **<0.01 (data are presented as mean +SEM).

Camptothecin, which stabilizes and activates p53, was applied to the stable cell lines alone or in combination with Sunitinib. Whereas Sunitinib affects the proliferation pathway, Camptothecin is known to affect both apoptosis and proliferation [33–35]. As expected, treatment with Camptothecin alone or in combination with Sunitinib highly increased apoptosis in all cell lines, whereas Sunitinib alone had no effect on apoptosis. Cells with Arg161Gln, Arg161Pro and Tyr98Asn showed the highest response to Camptothecin alone. Apoptosis was even increased with combined treatment in Arg161Gln and Tyr98Asn cells (Figure 9A).

An effect on cell proliferation was observed when the cells were treated with all three treatment strategies. With the exception of Arg161X, cell proliferation was decreased between 30-75% (Figure 9B). The response to the different treatments was similar for cell lines with missense mutation in the p53 binding domain and in the HIF1/2α binding domain.

## DISCUSSION

By investigating p53 expression in 262 ccRCC, we saw a relationship between p53 expression and the severity of *VHL* mutations. Our results are consistent with other studies showing that p53 expression was significantly lower in ccRCC than in other RCC subtypes [36–39]. The correlation between severe *VHL* mutations and negative or low p53 expression suggests a close relationship between loss of function of pVHL and disturbed p53 signaling in ccRCC.

As the location of a *VHL* missense mutation may specifically affect one of the many binding sites of pVHL, we focused on those missense mutations that alter the p53 binding site. Therefore, we selected four missense mutations identified in the p53 binding domain. The missense mutations were predicted to have different effects on pVHL stability, from highly destabilizing to neutral. [9]. As the p53 binding domain overlaps the ElonginC binding domain [17], the missense mutations in this region should affect pVHL interactions with p53 and/or HIF1/2α.

The *VHL* mutations Cys162Arg, Arg161Pro and Arg161X were unable to downregulate HIF1/2α at the protein level. This result was expected for the nonsense mutation Arg161X and for Arg161Pro due to its predicted destabilizing effect on pVHL stability but not for Cys162Arg which is predicted to be neutral. As it was demonstrated for the missense mutations Cys162Phe and Cys162Ala [40, 41], Cys162Arg also seems to impair ElonginC binding, thus leading to HIF1/2α accumulation. Mutant Arg161Gln remained only partly functional for HIF1/2α degradation but like *VHL*30 (wild-type), Leu158Val was able to fully downregulate HIF1/2α. Notably, Arg161Gln had a milder effect on HIF1α degradation than on HIF2α. In contrast to HIF1/2α, p53 expression was hardly affected by the *VHL* mutations. In a previous study it was shown that pVHL wild-type enhances p53 transcriptional activity for its downstream targets *p21* and *Bax* [17]. We hypothesized that *VHL* mutations could affect p53 activity rather than stability in our stable cell lines. Therefore, we analyzed the transcription levels of *TP53* and its downstream targets *p21*, *Bax* and *Noxa*.

The RNA levels of *TP53*, *p21* and *Noxa* were lower in all *VHL* mutant cell lines compared to those in RCC4 *VHL*30. *Bax* RNA levels were affected to a lesser extent. We also analyzed the RNA levels in normal kidney and ccRCC tissue. p53 signaling was similar in *VHL* wild-type ccRCC and in normal tissue. In *VHL* mutated tumors RNA levels of *TP53*, *p21*, *Bax* and *Noxa* were significantly lower than in tumors with *VHL* wild-type, which confirmed the results obtained from the cell lines.

Recent findings suggest complex regulation between p53 and HIF1α. p53 has been described to bind HIF1α [32] which negatively affects its stability and activity [31]. Our results demonstrated that *HIF1α* knockdown led to an upregulation of *TP53* and its downstream targets in both pVHL re-expressing and pVHL deficient RCC4. However, the RCC4 cell lines expressing *VHL* presented significantly higher RNA levels of *TP53*, *p21*, *Bax* and *Noxa* compared to the RCC4 pVHL-deficient cell lines, independently of *HIF1α* knockdown. This led us to the conclusion that pVHL is able to promote p53 signaling even in the presence of HIF1α.

To further prove the association of *VHL* mutation status with p53 signaling on the protein level in ccRCC tissue, we investigated the expression of the p53 downstream target p21 by immunohistochemistry on the same TMA that was stained for p53. p21 was expressed in only 22% of ccRCC. Similarly to p53, p21 expression was significantly lower in *VHL* LOF mutants compared to wild-type, thus supporting a potential role of pVHL's integrity in sustaining functional p53 signaling.

At least 12 p53 isoforms have been reported in the literature [42] and their abnormal expression was observed in a wide range of cancers, including RCC [43, 44]. The p53 antibody (clone DO-7) used in our study detects wild type p53, p53β and p53γ. Therefore, it cannot be excluded that tumors exclusively expressing other p53 isoforms may have been missed. However, mRNA expression data of the different p53 isoforms demonstrated that p53, p53β and p53γ are the main expressed isoforms in RCC [43, 44] suggesting minor impacts of p53 isoform expression on our results.

All our selected *VHL* mutations led to an attenuated apoptosis compared to *VHL* wild-type, but no significant change in cell proliferation was seen. Given their apoptosis deficiency, *VHL* mutant cells seem to have a survival advantage compared to *VHL* wild-type cells. However, cells with a missense mutation in the p53 binding domain were less apoptotic than cells with missense mutations affecting the HIF1/2α binding domain. Since *VHL* mutant cells affected apoptosis rather than cell proliferation, we treated the cells both with Camptothecin, a chemotherapeutic drug that stabilizes and activates p53 by inducing DNA damage and decreases cell proliferation and Sunitinib, a TKI which negatively influences cell proliferation and is used to treat ccRCC. Notably, Camptothecin was more effective than Sunitinib because it decreased cell proliferation and increased apoptosis in all *VHL* mutated cell lines. However, the missense mutation location and the affected binding domains may only partially explain the differing response to treatment. In a previous study, the *VHL* mutants S111R and S111C, which are located in the HIF1/2α binding domain showed a decrease in apoptosis compared to *VHL* wild-type via impairment of recruitment of coactivators p300 and Tip60 [21]. The loss of ability to recruit coactivators of p53 may explain the low apoptotic activity of our cell lines with *VHL* missense mutations located in the HIF1/2α binding domain.

**Figure 10:**
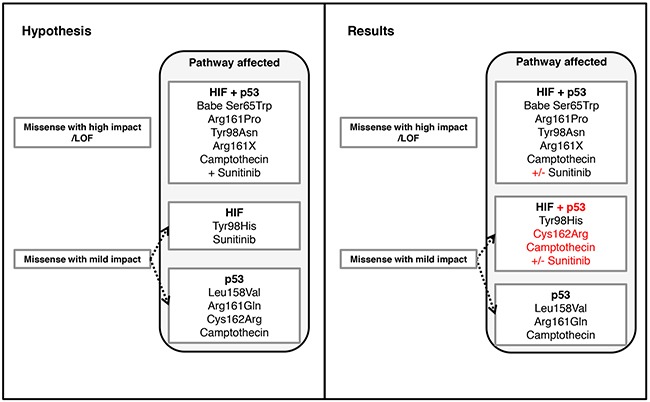
Comparison of predicted effects and own data of *VHL* mutations affecting the p53 and the HIF1/2α binding sites of pVHL and possible treatment strategies

In summary, we found an association of the *VHL* mutation type and p53 signaling, which was reflected by apoptotic deficiency. We also showed that pVHL integrity had a dominant effect over HIF1α downregulation in enhancing p53 transactivation suggesting disturbed p53 signaling is provoked by mutant pVHL rather than altered pVHL/HIFα axis. Notably, two *VHL* missense mutations in the p53 binding domain, Leu158Val and Arg161Gln, altered p53 signaling but retained HIF1/2α degradation function, thus confirming our hypothesis of differing effects of missense mutations on pVHL functions (summarized in figure 10) Our results may scrutinize the rationale of the systematic use of anti-angiogenic drugs, particularly for those metastatic ccRCC with *VHL* missense mutations that specifically affect non HIF1/2α binding sites. A systematic characterization of *VHL* mutations may help optimizing targeted therapy approaches for patients with metastatic ccRCC.

## MATERIALS AND METHODS

### Tissue Microarray and immunohistochemistry

A TMA [45] was stained using the mouse antibody p53 (clone DO-7, dilution 1:150, Dako A/S). As the tumors either showed hardly any or clearly more than ten positive nuclei, cores with up to five nuclei positive for p53 were grouped into “Low content” category and cores showing at least six positive nuclei were grouped into “High content”. The TMA was also stained and analyzed for p21 using the rabbit antibody p21 (C-19) (sc-397, dilution 1:50, Santa-Cruz Biotechnology) and scored following the same criteria used for p53.

### *In silico* selection of *VHL* mutations

We used the Site Directed Mutator *in silico* tool [46] to characterize *VHL* missense mutations in 360 FFPE ccRCC samples [47] which were re-reviewed by two pathologists (H.M., E.D.) according to the new WHO/ISUP grading system [48] (Table 2). The program calculates the thermodynamic change (ddG) occurring after modification of one amino acid according to the main chain conformation, solvent accessibility and hydrogen bonding class. The missense mutations were then classified as follows:

**Table 2:** Grading according to the new WHO/ISUP grading system and tumor stage of 360 ccRCC

ISUP Grade	N (%)	pT stage	N (%)
1	11 (3.1)	1	147 (40.8)
2	105 (29.2)	2	31 (8.6)
3	115 (31.9)	3	160 (44.4)
4	115 (31.9)	4	8 (2.2)
unknown	14 (3.9)	unknown	14 (3.9)

- ddg < -2.0 or >2.0: highly destabilizing or stabilizing referred as “high impact”

- -2.0 ≤ ddg < 2.0: (slightly) destabilizing, neutral, (slightly) stabilizing referred as “mild impact”

The binding domains of HIF1/2α (amino acid 67-117), p53 (aa 154-163) and Elongin C (aa 157-171) were assessed as described by Leonardi et al. [49]. We selected four mutations that were located in the overlap of p53 and EloC binding domains of pVHL (aa 157-163): three were predicted to have no or little impact on the protein stability (Leu158Val, Arg161Gln, Cys162Arg, mild impact) and one was predicted to highly destabilize pVHL (Arg161Pro, high impact). Controls located in the HIF1/2α binding domain (Tyr98His, Tyr98Asn) and one nonsense mutation (Arg161X) were also included.

### Knock down, transfection and transduction experiments

The pVHL deficient cell line RCC4 (expressing *VHL* Ser65Trp, highly destabilizing mutation) was kindly provided by W. Krek (ETH Zurich, Switzerland) and grown under conditions recommended by ATCC. This cell line has been authenticated as RCC4 plus vector alone (ECACC 03112702) in June 2016. A pcDNA3.1 vector encoding *VHL* wild-type [9] was used to generate the selected mutants with the Quick Change Lightning site-directed mutagenesis kit (Agilent technologies, United States). Subsequently, pcDNA3.1 was subcloned into pBabe vectors for transduction in mammalian cells.

pBabe empty vector and vectors containing the *VHL* wild-type or the mutant *VHL* sequences were transfected into Platinum-A Retroviral Packaging Cell Line (Cell Biolabs, United States) for viral pseudo-particles production according to the X-tremeGENE 9 DNA Transfection Reagent 3:1 protocol (Roche Diagnostics, Switzerland). The viral supernatant was collected and applied to RCC4 for transduction following the manufacturer's recommendations. Polyclonal batches of the transduced RCC4 were then selected with constant concentration of 4μg/mL puromycin for four weeks and then maintained at 2μg/mL for cell culture.

Knockdown of *HIF1α* in RCC4 using short hairpin RNA (shHIF1α NM_001530.x_1048s1c1, Sigma) was performed as described previously [50].

### Western blot (WB)

WB was performed as described elsewhere [50]. Detailed information of the antibodies used for WB analysis is listed in Table 3.

**Table 3:** Antibodies used for Western blot analysis

Antigen	Dilution	Antibody	Provider
pVHL	1:1000	S2-647	BD Biosciences
p53	1:2000	Ab1101	Abcam
HIF1α	1:1000	NB100-479	Novus Biologicals
HIF2α	1:1000	PAB12124	Abnova
CA-IX	1:2000	M75	J. Zavada, Prague, Czech Republic
Glut1	1:1000	07-14-01	Millipore
Actin	1:2000	MAB15-01	Millipore
Goat anti-rabbit	1:1000	7074	Cell signaling
Goat anti-mouse	1:2000	Ab672	Abcam

### RNA extraction and quantitative PCR

RNA was extracted from cultured cells or from two core punches of FFPE samples using Maxwell® 16 LEV simplyRNA and RNA FFPE Purification Kit (Promega corporation,USA). RNA was reversely transcribed into cDNA using the High-Capacity cDNA Reverse Transcription Kit. RNA levels were determined by quantitative PCR using TaqMan® Gene Expression Assay on the ViiA7 Real-Time PCR System instrument (Applied Biosystems, United States). The probes used were Hs00184451 (*VHL*), Hs00153340 (*TP53*), Hs00355782 (*p21*), Hs00180269 (*Bax*), Hs00736699 (*Noxa*), Hs00936377 (*HIF1α*) and Hs01026146 (*HIF2α*). The results were normalized to *PPIA* (Hs99999904) RNA level in each sample [51]. The results are shown as Relative Quantity (RQ).

### Proliferation and apoptosis assays

A colorimetric Cell Proliferation ELISA BrdU assay (Roche diagnostics, Switzerland) and a Caspase-Glo® 3/7 Assay System (Promega corporation, United States) was used to investigate the proliferative and apoptotic behavior of the established cell lines, respectively.

### Drug treatment

Camptothecin and Sunitinib (Selleck Chemicals, USA) were diluted in DMSO at 0.05 μM and 10 μM concentration, respectively, and applied to the cells alone or in combination for 48h at a final concentration of 0.3% DMSO following the manufacturer recommendation. The vehicle control with 0.3% DMSO was referred to as “mock”, and “mock *VHL*30” was used as a reference.

### Statistics

Two-tailed Chi square, Fischer's exact test and Student's T-tests were performed using the program Graphpad prism 5.04 for Windows (GraphPad Software, San Diego California USA, www.graphpad.com).
